# Supplementary material for: A spatial map of hepatic mitochondria uncovers functional heterogeneity shaped by nutrient-sensing signaling
Source: bioRxiv. 2023 Oct 26:2023.04.13.536717. Originally published 2023 Apr 13. Preprint. [Version 2] doi: 10.1101/2023.04.13.536717 (PMC10274915; doi:10.1101/2023.04.13.536717)
Supplement: Supplement 6 — Figure S1. Comparative proteomics of spatially sorted hepatocytes. (A) Principal component analysis (PCA) and frequency histogram of spatially sorted hepatocytes analyzed with mass spectrometry. (B) Correlation Matrix Heatmap of proteomics data. (C) Pie chart depicting the percentage of PP, PC, and UZ proteins based on p-value (0.05). (D) Volcano plot showing the PC to PP log2 fold-change (x-axis) and the −log10 p-value (y-axis) for identified proteins. (E and F) GO enrichment analysis of the proteomics data in the spatially sorted cell. Figure S2. Immunofluorescence of representative PP and PC mitochondrial proteins. (A) Confocal image of the liver lobule from Mito-Dendra2 mice (green) stained with E-cadherin to label PP regions (yellow) and Aldh1b1, a PP mitochondrial protein (magenta). Magnified insets show the overlay of E-cadherin and Aldh1b1 only. Scale bar: 15 µm. (B) Confocal image of the liver lobule from Mito-Dendra2 mice (green) stained with E-cadherin to label PP regions (yellow) and Oat, a PC mitochondrial protein (magenta). Magnified insets show the overlay of E-cadherin and Oat only. Scale bar: 15 µm. Figure S3: E-cadherin-positive hepatocytes display higher membrane potential. (A-B) Intravital microscopy of the hepatic lobule in Mito-Dendra2 mouse labeled with TMRE (red), and E-cadherin (white). (A) Low magnification of the hepatic lobule. Scale bar: 30 μm. (B) Close up on the PP-PC axis. Scale bar: 20 μm. Figure S4. Lipid synthesis is pericentral in the murine liver. (A) Confocal image of a liver section labeled with Lipidtox (lipid droplets; red) and phalloidin (actin; cyan), showing the non-uniform distribution of lipid droplets across the PP-PC axis. (B) Representative immunoblot and quantification of acetyl-CoA carboxylase 1 (ACC1, Acaca) relative phosphorylation (S79) in spatially sorted hepatocytes. Bar graph shows five independent experiments. (C) RNA levels of key lipogenesis enzymes in spatially sorted hepatocytes. Bar graph shows three ind [file NIHPP2023.04.13.536717v2-supplement-6.pdf]

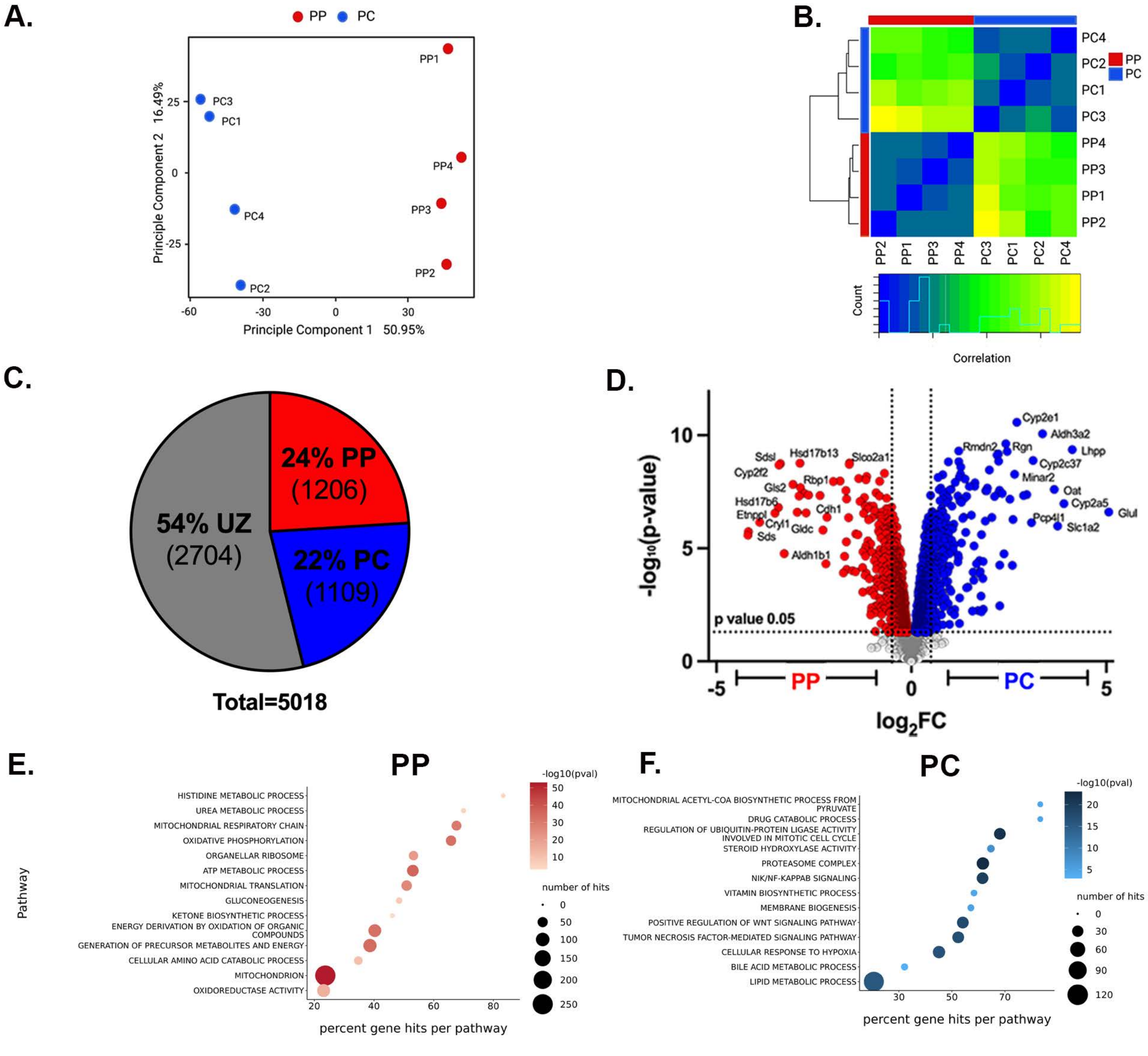

Fig S1 Comparative proteomics of spatially sorted hepatocytes

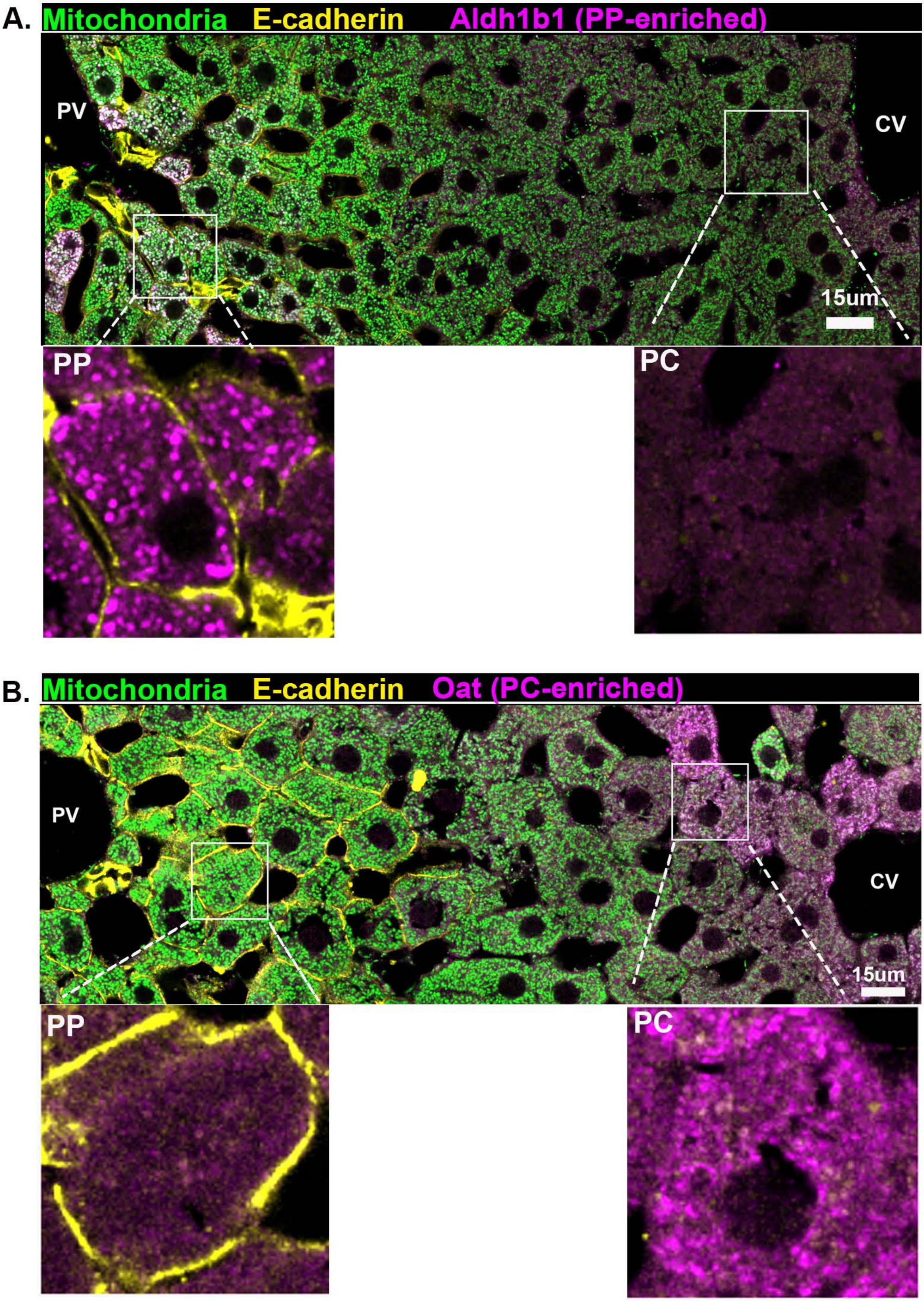

Fig S2. Immunofluorescence of representative PP and PC mitochondrial proteins

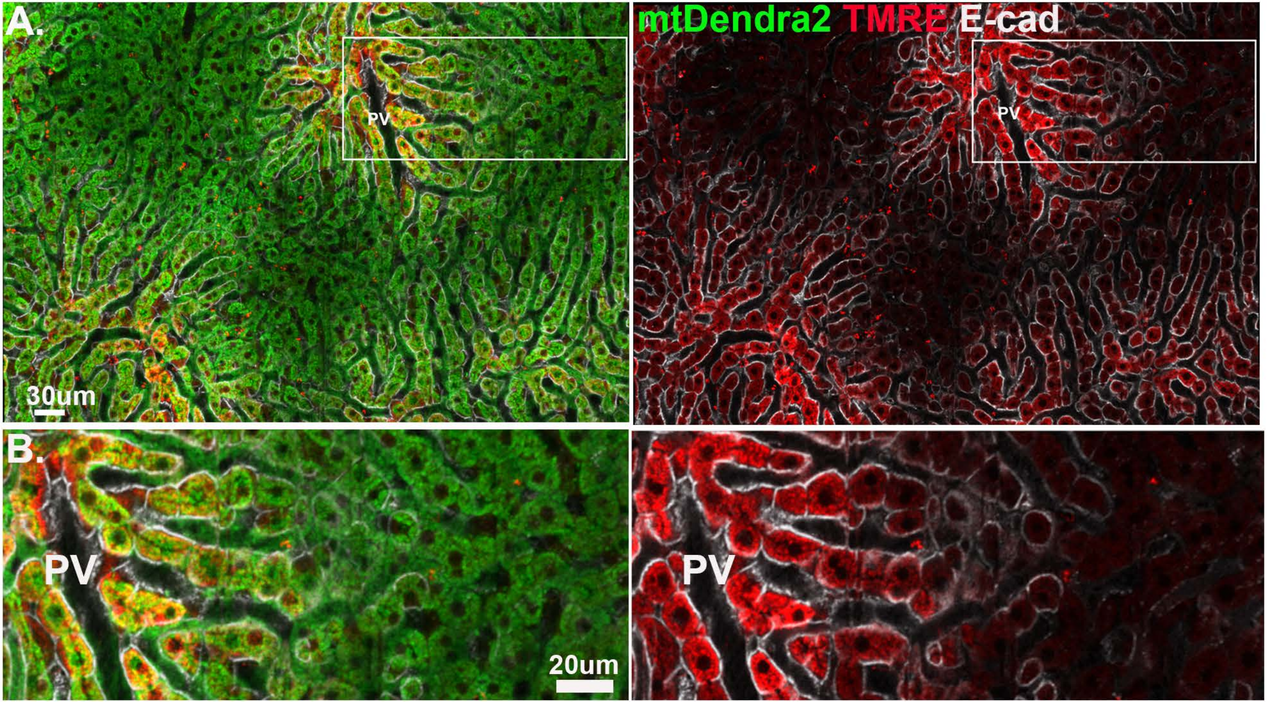

Fig S3. E-cadherin positive hepatocytes display higher membrane potential

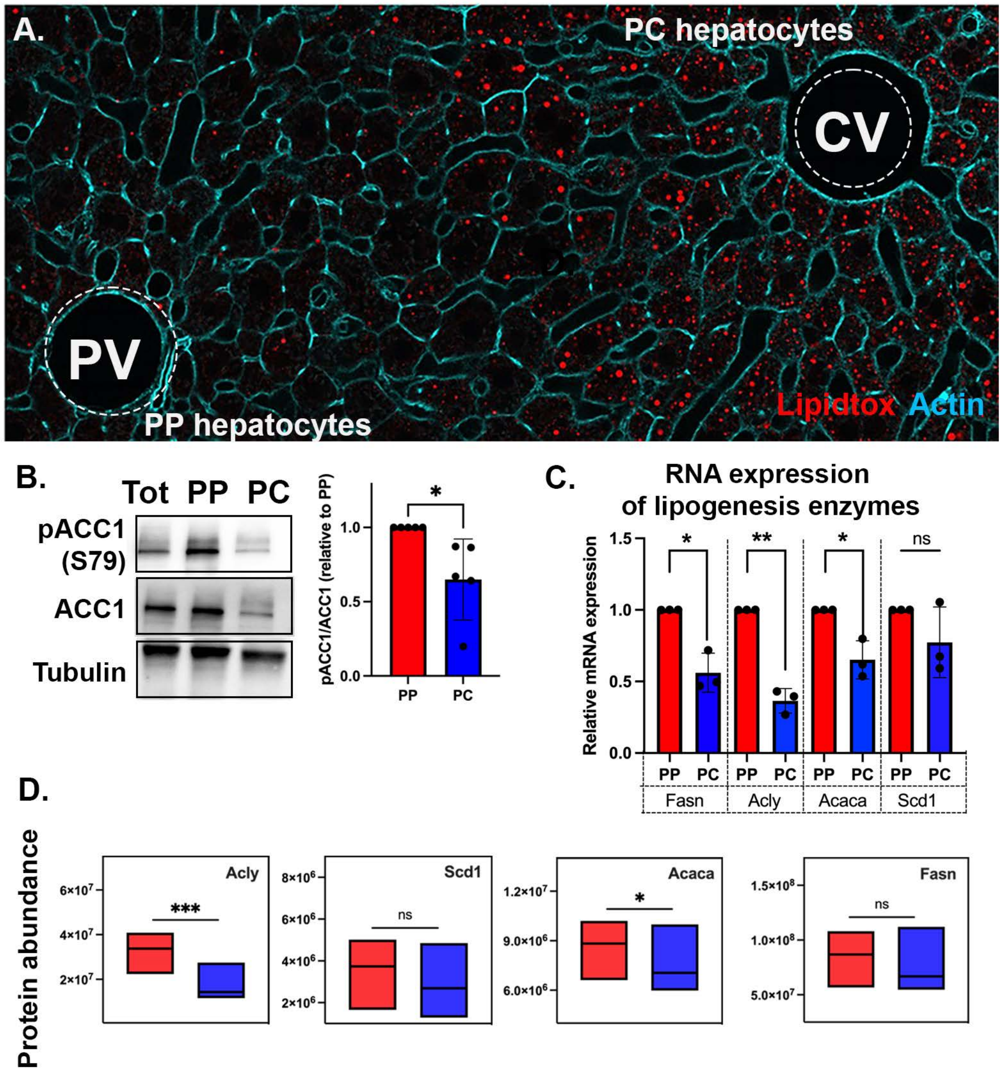

Fig S4. Lipid synthesis is pericentral in the murine liver

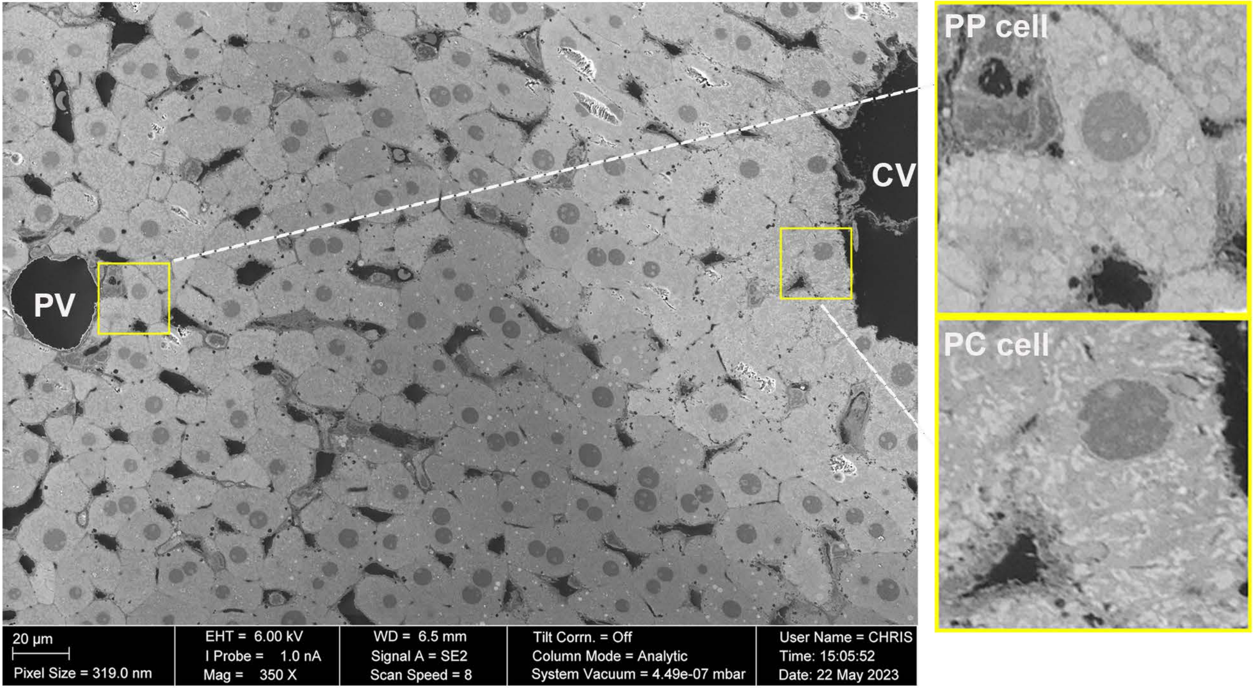

Fig S5. Scanninig Electron Microscopy of the lobule highlighting regions of interest selected for FIB-SEM

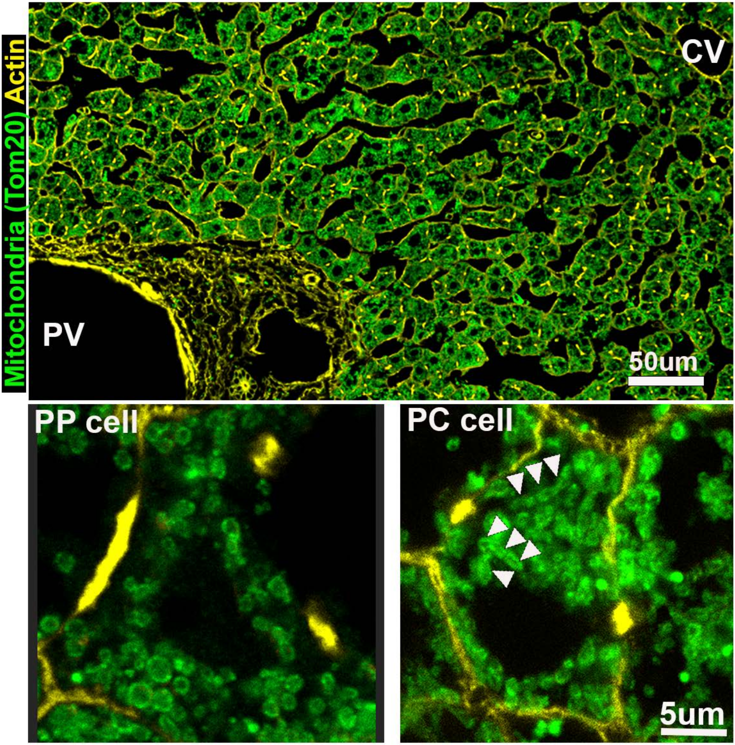

Fig S6. Mitochondrial morphologies are conserved in the human liver

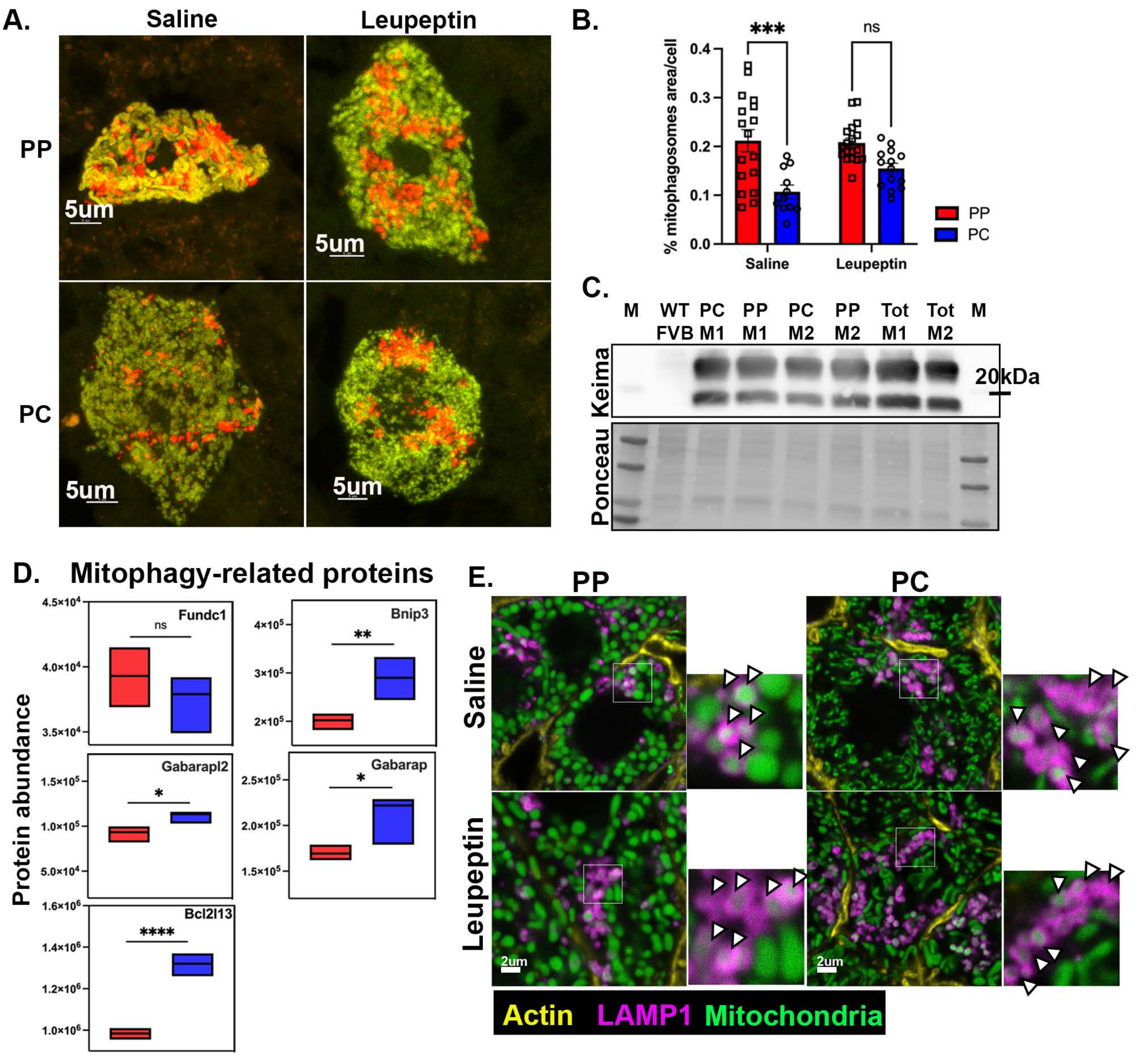

Fig S7. PC hepatocytes display a higher mitophagy flux

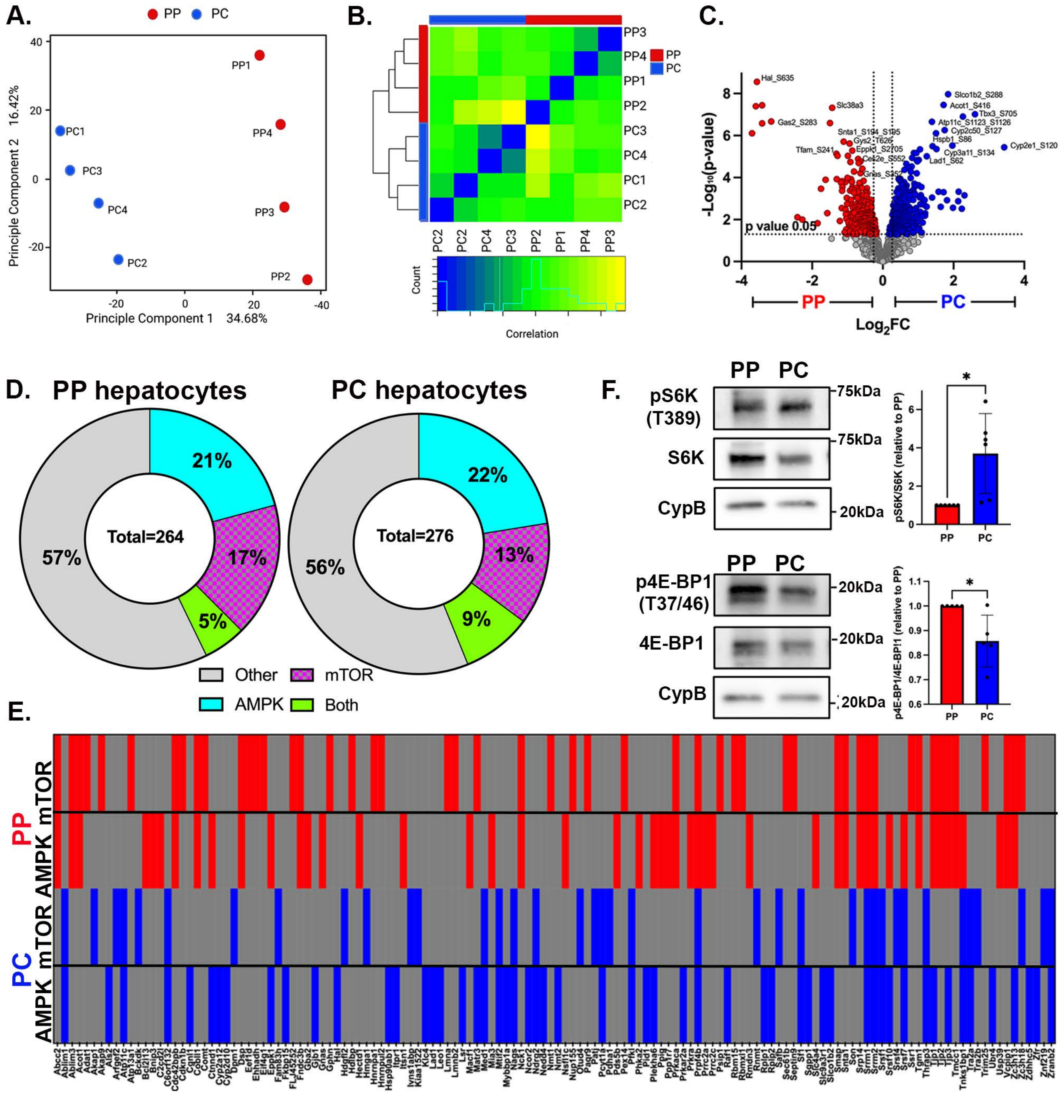

Fig S8 Hepatocyte phosphoproteome

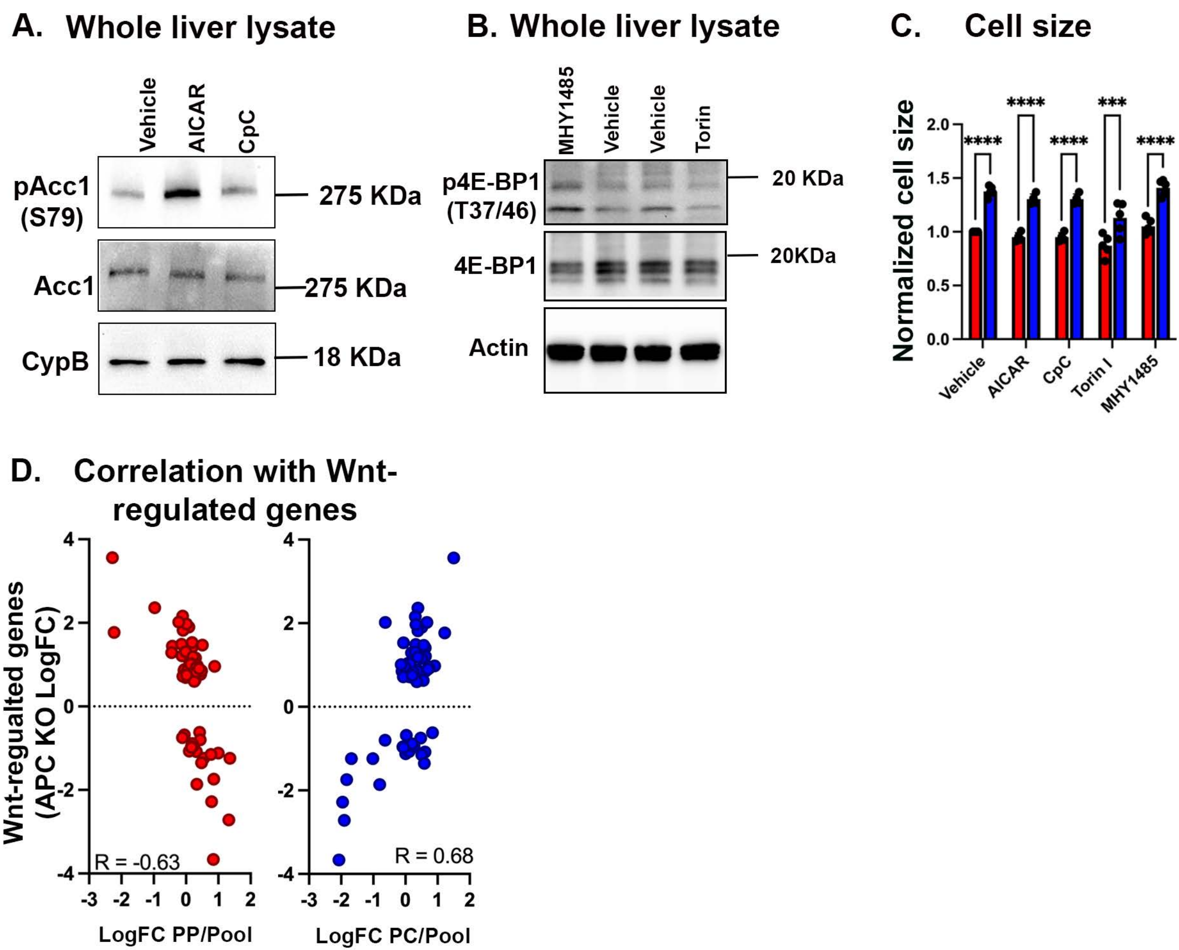

Fig S9. Nutrient sensing signaling regulates mitochondrial heterogeneity
